# Development of ternary nanoadsorbent composites of graphene oxide, activated carbon, and zero‐valent iron nanoparticles for food applications

**DOI:** 10.1002/fsn3.1080

**Published:** 2019-07-29

**Authors:** Mahsa Bagheri, Seid Mahdi Jafari, Mohammad H. Eikani

**Affiliations:** ^1^ Department of Food Science and Technology, Sari Branch Islamic Azad University Sari Iran; ^2^ Department of Food Materials and Process Design Engineering Gorgan University of Agricultural Sciences and Natural Resources Gorgan Iran; ^3^ Department of Chemical Industries Iranian Research Organization for Science and Technology Tehran Iran

**Keywords:** active carbon, graphene oxide, nanoadsorbents, nanocomposite, nZVI, separation

## Abstract

In this study, a ternary nanocomposite comprising graphene oxide and carbon loaded with zero‐valent iron nanoparticles was developed as a promising nanoadsorbent, especially for polyphenols available in food industry by‐products. The fabricated nanoadsorbents were characterized in terms of structural, morphological, and chemical attributes. Zero‐valent iron nanoparticles (nZVI) were produced by a modified method leading to the formation of nanoparticles below 50 nm. Also, active carbon was transformed to a needle‐like shape instead of its native shape so that the active surface area was drastically increased which favors the higher adsorption process. Moreover, the space between graphene oxide sheets was enhanced by ultrasonication so that more active carbon and nZVIs could be oriented between these sheets. Finally, the FTIR and Raman data demonstrated the formation of O‐H stretching groups and a D/G value of 0.85 corresponding to the maintenance of a desired structure of the graphene oxide sheets, respectively. To summarize, the developed nanocomposites can be employed as a promising tool for the adsorbance of food and beverage industry by‐products, especially polyphenols.

## 1 INTRODUCTION

Food processing factories generate a lot of by‐products annually which are detected by their chemical oxygen demand (COD), biological oxygen demand (BOD), total suspended solids (TSS) plus the pathogens, and bioactive components (Assadpour, Jafari, & Esfanani, 2017; Falahati, Baghdadi, & Aminzadeh, 2018). These highly valuable by‐products can be recovered and used as natural food ingredients to minimize the consumption of synthetic food‐grade additives. Furthermore, the secondary metabolites like polyphenols and carotenoids plus high molecular components of plant cells have several benefits, such as antioxidant, antimicrobial as well as UV‐protecting features, that can be employed as natural nutraceuticals and food‐grade bioactives to improve the nutritional and sensorial features as well as extending the shelf life of food products. The bioactive compounds found in food by‐products may also be employed in the production of functional food products. As an example of food processing by‐products, over 3,200 tons of phenolic compounds are wasted during olive oil production in the world (Schieber, 2017). According to the recent articles, some of the principal by‐products of food processing include phytosterols, polyphenols, dietary fibers, vitamins and minerals, pigments, peptides, etc. (Offiah, Kontogiorgos, & Falade, 2018; Rahmanian, Jafari, & Galanakis, 2014; Ralla et al., 2018).

Zero‐valent iron (ZVI) is applied as an active agent for fixation and reduction of contamination, especially in case of polyphenols and food processing wastewater (Kowalski & Søgaard, 2014). Recently, zero‐valent iron nanoparticles (nZVI) have captured the attention of both industry and academia due to their special surface properties, permeability, and high reactivity owing to their extra small size. However, they are susceptible to oxidation as they interact with water molecules; thus, their performance is hindered. Also, these nanoparticles have a high tendency to agglomerate, and as a result, their performance is significantly minimized. As a solution, surface protection of these nanoparticles by recyclable and green materials, such as active carbon, resin, bentonite, and silica, is proposed (Li, Ai, & Jiang, 2016).

Activated carbon (AC) is an efficient material known for its significant adsorption performance, surface area, and cost efficiency. The aforementioned features make AC a promising material for the adsorption of several grafting agents, such as phenolic compounds (Kumar & Jena, 2016; Yangui & Abderrabba, 2018), heavy metals in waste water treatment (Kołodyńska, Krukowska, & Thomas, 2017; Nayak, Bhushan, Gupta, & Sharma, 2017), dyes (Tseng, Wu, & Juang, 2003), and other compounds. Accordingly, the adsorption capacity of AC for aromatic compounds depends on some factors including the physical nature of the adsorbent, functional groups, pore size and ash level, the structure of the adsorbate, p*k*_a_, functional groups of the adsorbate, size and molecular weight, and finally the solution circumstances like pH, ionic strength plus the amount of the adsorbate. AC is an efficient material for applications in nanocomposites used for better adsorption results (Mohanta & Ahmaruzzaman, 2018).

Graphene is another interesting molecule for the adsorption of several compounds which is composed of a single layer of carbon atoms oriented in a hexagonal lattice. Owing to the outstanding attributes of graphene like a high rate of heat transfer, resistance to fracture, high electrical conductivity, and large surface area, it can be applied to protect nZVI. It has the benefit of cost efficiency, also in comparison with multiwalled carbon nanotubes and active carbon, graphene sheets have a high surface‐to‐volume ratio and therefore have more growing sites to be exposed to nZVI. Thus, the high rate of conductivity is beneficial for the electron transfer between the graphene and contaminants. With the packing of the nZVI between the graphene sheets, new physicochemical properties are obtained as well as creating stable structures. Moreover, with the placement of these nanostructures in an external magnetic field, the separation process would become simple (Fan et al., 2016; Jabeen, Kemp, & Chandra, 2013; Liu et al., 2016a; Lv et al., 2014; Xing, Xu, & Wang, 2016; Yang, Tian, Zhang, Guo, & Yan, 2015; Zhang, Dwivedi, Chi, & Wu, 2010).

The purpose of this study was to develop nanoadsorbents with the potential applications in the food industry for the first time based on ternary composites of graphene oxide with active carbon, and then, nZVI was placed between the sheets. Furthermore, the physical, morphological, and chemical attributes of the developed nanocomposite adsorbents were examined by using FE‐SEM, AFM, EDS, Raman spectroscopy, and FTIR plus Mapping tests and the results were analyzed.

## 2 MATERIALS AND METHODS

### 2.1 Materials

Graphite, active carbon, NaBH_4_ (98%, Acros), ferrous sulfate (FeSO_4_.7H_2_O), potassium permanganate (KMnO_4_), sodium nitrate (NaNO_3_), hydrogen peroxide H_2_O_2_ (30 wt%), polyvinylpyrrolidone (PVP), sulfuric acid (H_2_SO_4_, 98%), hydrochloric acid (HCl, 35%–37%), and ethanol (absolute) were of all analytical grade and prepared from Merck Company. Milli‐Q water was implemented in the entire production stages.

### 2.2 Synthesis of nZVIs

nZVIs were prepared from the mixture of ferrous sulfate (FeSO_4_.7 H_2_O) 0.05 M and sodium borohydrate 0.2 M. Initially, 0.05 M of ferrous sulfate (FeSO_4_.7 H_2_O) was placed in a three‐necked flask which had an inlet for N_2_ gas and an outlet for H_2_ from the middle outlet of the flask. NaBH_4 _(0.2 M) was suddenly added to the middle of the flask with short intervals (5–15 s) and as the extra content of the hydrogen gas is released during the interaction, the solution was stirred at 400 rpm. After 1 hr, the black‐colored solution was centrifuged and rinsed for three times with double‐distilled water and alcohol in the ratio of 1:1 and centrifuged again. Finally, the yield was dried by N_2_ gas and kept in a vacuum desiccator.

### 2.3 Graphene oxide Preparation

The synthesis procedure was done according to the method of Hummer (Hummers Jr and Offeman, 1958). First, graphite (5–7 micron, 2 g) and sodium nitrate (1.5 g) were mixed in a flask placed in an iced bath, and then, sulfuric acid (98%, 150 ml) was added during stirring. In the next step, 9 g of potassium permanganate was added, and these circumstances were maintained for 2 hr, and finally, the ice bath was discarded. After storing the mixture for 5 days at ambient temperature, 6 ml of hydrogen peroxide was added to the solution to neutralize potassium permanganate. Subsequently, the solution (250 ml) was mixed with sulfuric acid (98%, 7.5 ml) and hydrogen peroxide (30% wt, 4.17 ml), and double‐distilled water was added until a yellowish suspension emerged. The precipitate was dialyzed for 5 days at a neutral pH in order to eliminate the remaining ions. The resulted graphene oxide was then treated with ultrasonication and dried under vacuum condition when it demonstrated a gray color.

### 2.4 Composite nanoadsorbent preparation

The ternary nanocomposites were manufactured by the reduction of ferrous sulfate in combination with active carbon and graphene oxide (GO/AC‐nZVI). Graphene oxide in deionized water was dispersed in an ultrasonic bath during 3 hr and added to active carbon powder. In the next step, PVP (0.1716 g) and ferrous sulfate were added to the solution under nitrogen gas and stirred for 3 hr. Sodium borohydrate (2.5 g/50 ml) was added dropwise into the solution, and the stirring process continued for 5 hr. According to Equation (1), ferrous ions are reduced into ZVI:

(1) $${2Fe}^{2+}+{BH}_{4}^{-}+{2H}_{2}O={2Fe}^{0}+{BO}_{2}^{-}+{2H}_{2}+{4H}^{+}$$

The black precipitate obtained from centrifugation was washed three times with ethanol, stored, and dried under nitrogen environment. The entire composite synthesis steps were done in a 500‐ml reactor flask under the injection of nitrogen gas to completely prevent the oxidation process of nZVI. Moreover, a water bath was applied to maintain the temperature at 25°C. The control sample was prepared according to the above‐mentioned procedure without the addition of graphene oxide and PVP. Final ternary nanocomposite samples were prepared according to Table 1.

**Table 1:** Formulation of the nanocomposite samples

Number	Sample code	Activated Carbon (mg)	Graphene Oxide (mg)	FeSO_4_.7H_2_O (mg)
Control	C	500	‐	‐
1	C_300_‐G_600_	300	600	789
2	C_600_‐G_300_	600	300	789
3	C_400_‐G_400_	400	400	789

### 2.5 FTIR and Raman spectra

A Bruker Tensor 27 FTIR spectrophotometer (Germany) was employed to detect the FTIR spectra of the samples. Accordingly, KBr pellets and samples were dried in a vacuum oven at 353 K for 12 hr to reduce the impact of moisture on the spectrum, and the FTIR spectrum was scanned in transition mode from 400 to 4,000 cm^−1^.

Raman spectra were obtained in the 600–3,500 cm^−1^ range by a Bruker Optics model Senterra, employing 532‐nm wavelength incident laser light and 20 mW power. The high throughput of this instrument permitted the use of very low energy densities at the sample.

### 2.6 Field emission scanning electron microscopy (FE‐SEM)

Field emission scanning electron microscopy (FE‐SEM; MIRA2 LMV‐TESCAN) operating at 15 kV was implemented to view the morphology of the nanocomposite plus energy‐dispersive X‐ray spectra (EDS) as well as elemental mapping.

### 2.7 Atomic force microscopy (AFM)

The surface and height profile of samples were analyzed by atomic force microscopy (AFM, Digital Instruments NanoScope V) after placing a drop of the sample onto a surface of mica in tapping mode.

## 3 RESULTS AND DISCUSSION

### 3.1 Morphology and microstructure of nanoadsorbents by FE‐SEM analysis

#### 3.1.1 Zero‐valent iron nanoparticles (nZVI)

**Figure 1:**
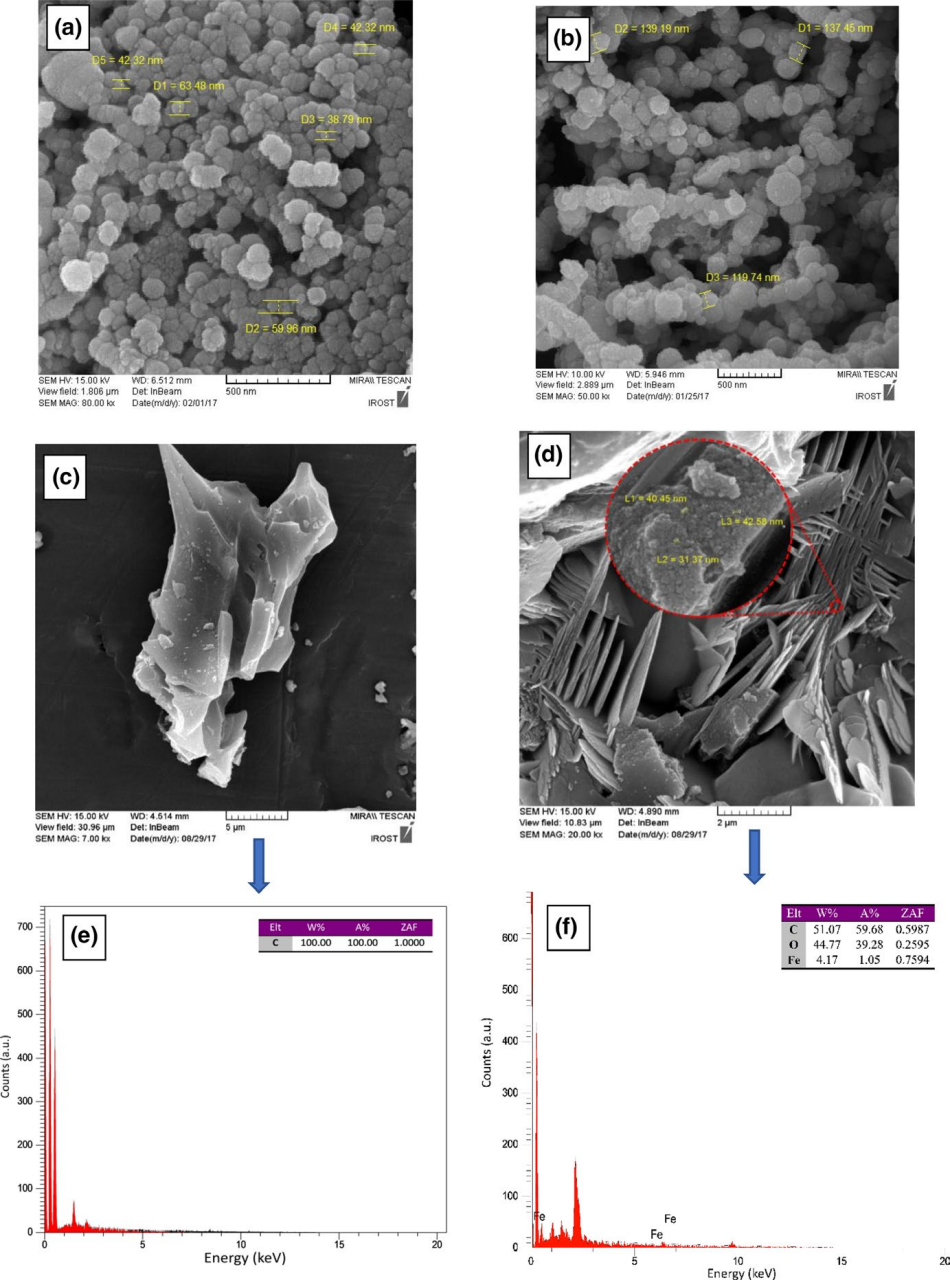
Field emission scanning electron microscopy (FE‐SEM) images of nZVI (a, b), AC (c), and composite of AC + nZVI (d), energy‐dispersive X‐ray spectroscopy (EDX) pattern of AC (e), and composite of AC + nZVI (f)

The generated ZVI nanoparticles were examined by FE‐SEM (Figure 1a and b). The results suggested that the size of prepared particles is within the nanoscale. When the produced nanoparticles were added dropwise to the solution, their size was higher than 100 nm. However, by adding the entire sodium bromate to the solution all at once, the size of the nanoparticles was lowered to 50 nm; probably it takes place as a result of having no time for the particles to grow. Bae, Gim, Kim, and Hanna (2016) studied the effect of sodium bromate addition on the size of the fabricated nZVI. They reported that without the addition of sodium bromate, chain‐shaped aggregates of ZVI were obtained (60–100 nm); however, by adding the 100 mM sodium bromate, the size of the nZVIs was drastically reduced to 50 nm and kept the same morphology (spherical shape). They suggested that the size reduction was mainly due to oxidative dissolution of nZVI or by chemical etching reaction. Similarly, Akhavan, Assadpour, Katouzian, and Jafari (2018) reported that by adding sodium borohydride, over 80% of the formed nanoparticles are below 100 nm and 50% are less than 50 nm with respect to the acquired SEM images. As can be seen in Figure 1a and b, the fabricated nZVIs tend to aggregate since they are colloidal in nature. Also, they are able to adhere to different types of surfaces like soil and sediment. Some studies have also been conducted for the dispersion sake of nZVIs such as the usage of water‐soluble starches as a stabilizer for the nanoparticles (He & Zhao, 2005) plus carboxymethyl cellulose for nZVI (He & Zhao, 2007).

#### 3.1.2 Active carbon and its nanocomposites with nZVI (control sample)

Active carbon was analyzed using the FE‐SEM device, and results are shown in Figure 1c. As can be seen, active carbon bodies are aggregated and disordered large sheets. The combination of active carbon with ZVI in the control sample transforms the active carbon morphology to rod‐shaped structures and nZVI is dispersed uniformly on the active carbon with the size of 40 nm (Figure 1d).

According to the results of Juang et al. (2018) who fabricated Fe_3_O_4_/activated carbon nanocomposites for the treatment of wastewater, the developed nanocomposites represented rod‐shaped active carbons with a high surface area, and the Fe_3_O_4_ particles were dispersed uniformly on the active carbon surface which approves the results of this study. Moreover, Liu et al. (2016b) developed nZVI‐activated carbon composites for the formation of an efficient microgalvanic cell as a reduction agent of nitrate. The SEM analysis revealed that chain form aggregates of nZVI are deposited mostly on the surface of the active carbon bodies which promotes the process of microelectrocatalysis.

The evaluation of the control sample in Figure 1d suggested that nZVI exists both on the external and on the internal surfaces of active carbon, while the study conducted by Zhu, Jia, Wu, and Wang (2009) reported that most of the nZVIs are placed in the internal cavities and fractures. This trait is of great importance as it leads to the loss of nZVI and thus the reduction in the adsrobtion process. On the other hand, energy‐dispersive X‐ray (EDX) spectra shown in Figure 1e and f clearly show the presence of Fe in composited of active carbon and nZVI.

#### 3.1.3 Ternary nanocomposites of graphene oxide, active carbon, and nZVI

**Figure 2:**
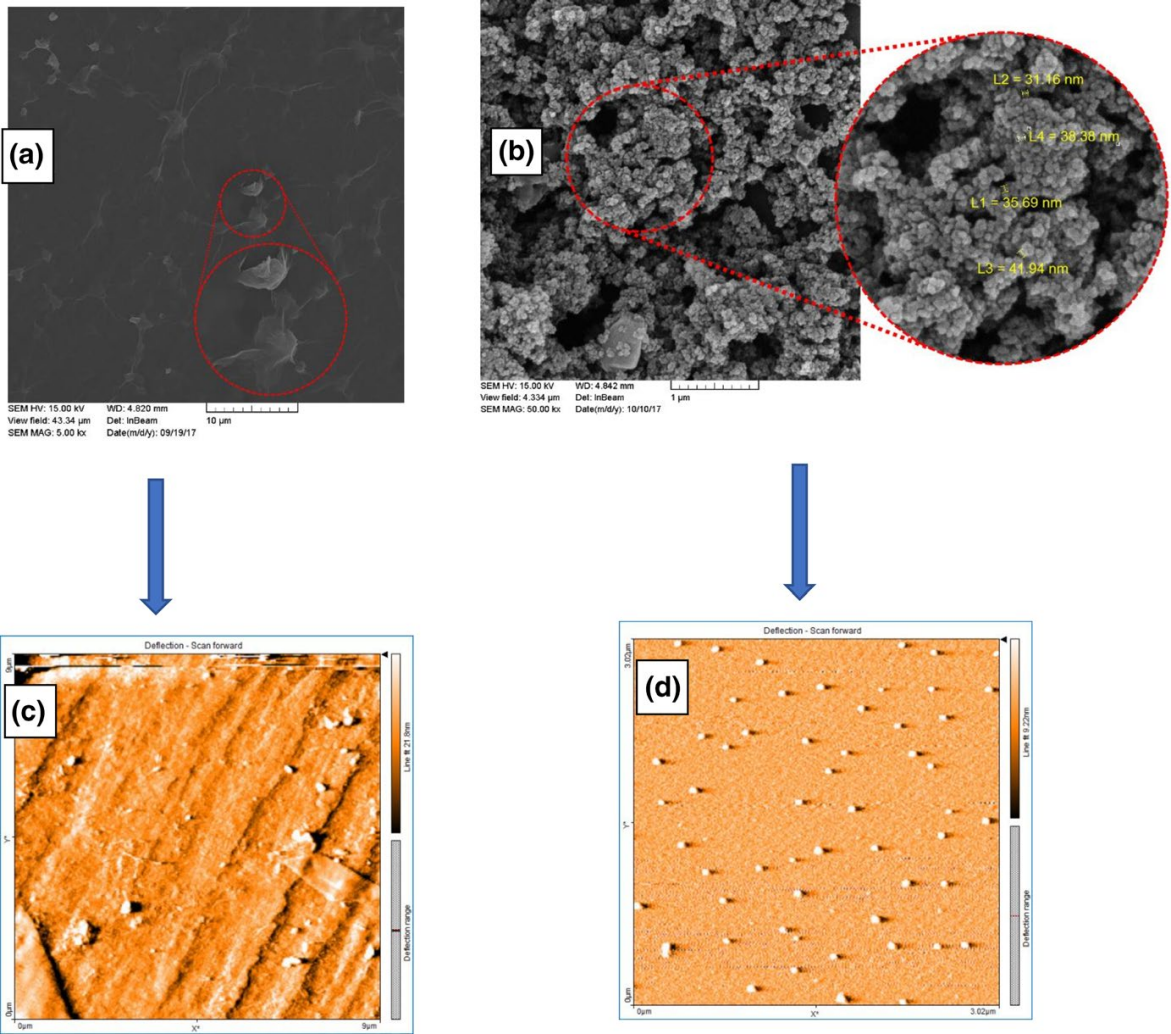
Field emission scanning electron microscopy (FE‐SEM) images of GO (a) and final nanocomposite sample C_300_‐G_600 _(b) compared with contact mode AFM analysis of GO (c), and final nanocomposite sample C_300_‐G_600 _(d)

The FE‐SEM image of graphene oxide (GO) as shown in Figure 2a illustrated that the graphene oxide layer is dispersed and bears a shrinked shape. The chief pattern of graphite was shaped via the addition of oxygen covalent bonds and placement of carbon atoms with the hybridization of sp3. The shrinkage which is present in the image illustrates the oxygen‐containing functional groups of carboxyl and hydroxyl on the surface of graphene oxide.

**Figure 3:**
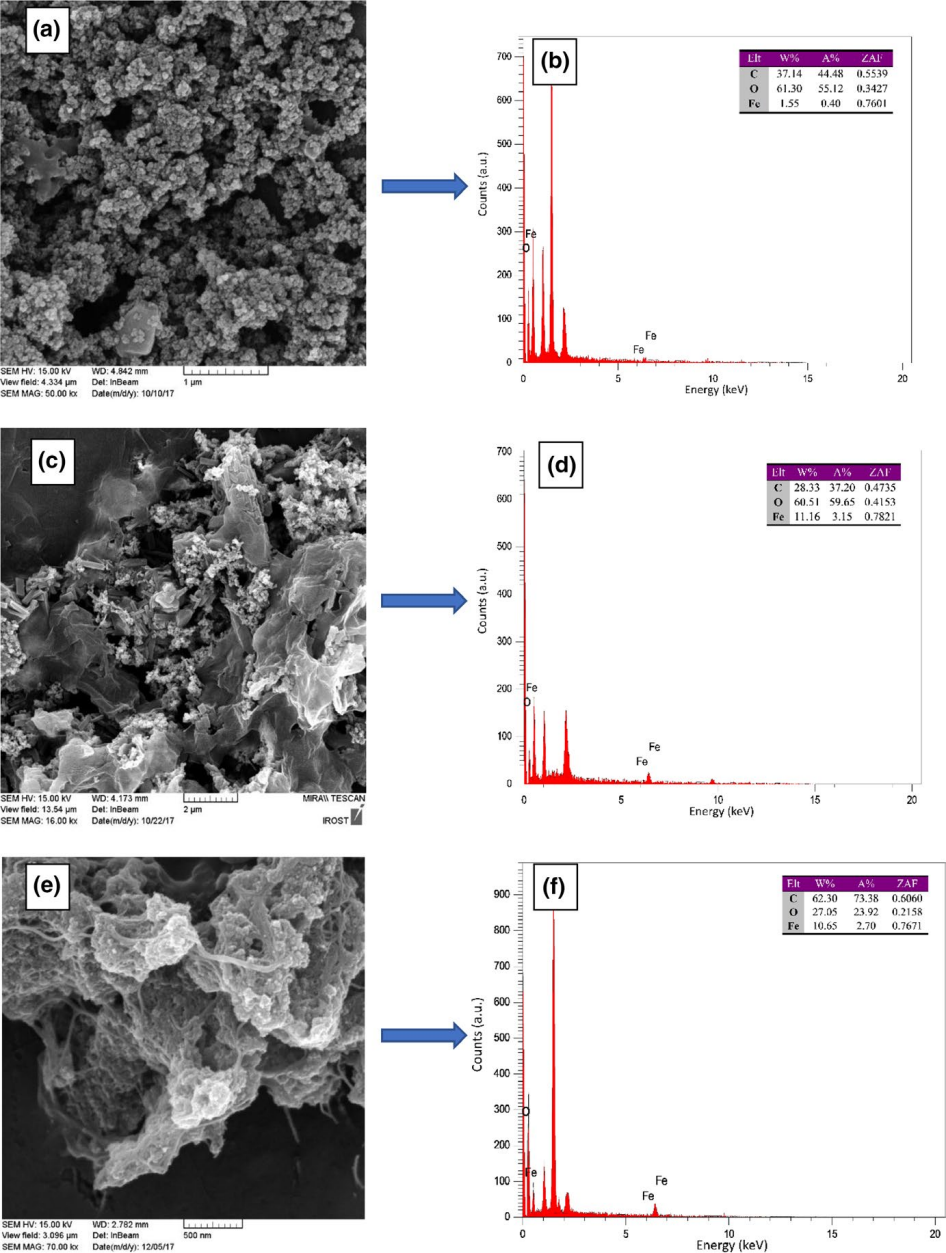
Field emission scanning electron microscope (FE‐SEM) images of final nanocomposite samples: C_300_‐G_600 _(a), C_600_‐G_300_ (c), and C_400_‐G_400_ (e). Energy‐dispersive X‐ray spectroscopy (EDX) pattern of final nanocomposite samples: C_300_‐G_600 _(b), C_600_‐G_300_ (d), and C_400_‐G_400_ (f)

Figure 3a, c and e represents the FE‐SEM images of Go/AC/nZVI ternary nanocomposite. Following the process of graphite oxidation, different functional groups like hydroxyl and epoxy entered the active carbon, and as a result, active carbon was simply mixed with graphene oxide which can be attributed to the presence of these functional groups on the surface of active carbon and graphene. In Figure 3a, the concentration of graphene oxide was higher than active carbon (sample: C_300_‐G_600_), and an aggregated cluster form of nZVI was observed, and graphene sheets were not exposed, whereas in the conditions that active carbon had a greater concentration than graphene (Figure 3c; sample: C_600_‐G_300_), the structure of graphene oxide and the rod‐shaped active carbon was clearly visible. However, in the samples which had lower extent of graphene oxide, more active carbon and nZVI structures were detected due to the considerable spaces between the graphene oxide sheets. By comparing the FE‐SEM images of the treatments at equal concentrations of graphene oxide and active carbon (Figure 3e; sample: C_400_‐G_400_), an intermediate architecture between the mentioned arrangements was formed. Accordingly, Li, Pan, Nie, Liu, and Sun (2012) fabricated reduced graphene oxide (RGO) plus activated carbon (AC) composites and according to the SEM prepared images, composites showed a sandwich structure that reduced the aggregation of AC particles and supported more space to accommodate ions during electrosorption.

### 3.2 Atomic force microscopy (AFM) results of ternary nanoadsorbents

The analysis of SEM images of ternary nanocomposites of Go/AC/nZVI presented an intact, clustered‐shape composite in Figure 2b. As can be seen in Figure 2d, for the relevant AFM image of the same sample (C_300_‐G_600_), the observed ordered structure is homologous to the SEM image. The AFM image of graphene oxide (Figure 2c) demonstrated that the formation of the thin‐layer graphene oxide can be observed. Accordingly, by comparing Figure 2a and b, the SEM image reveals a shrinked surface structure which is better observed in the AFM image as the dark spots demonstrate the indented surfaces, and the surface topography is clear. The observed concavities and convexities in the image indicate the shrinkage morphology of the FE‐SEM image of graphene oxide. In a similar study Maddinedi, Mandal, Vankayala, Kalluru, and Pamanji (2015) fabricated graphene oxide sheets by using *Terminalia chebula* extract. The FE‐SEM images of normally prepared GOs and those prepared with the extract illustrated stacked layers with a roughness which exists due to the oxidation of sheets. Moreover, WooáLee and BináKim (2011) reported a uniform distribution of nZVIs attached to the graphene nanosheets fabricated by the modified Hummer's method, and the thickness of the iron nanoparticles was 10.5 nm. They confirmed that the presence of the graphene oxide sheets protects nZVI from oxidation as well as reducing the agglomeration of nZVI and rendering the final nanocomposite water dispersible.

### 3.3 Raman and FTIR spectra results of ternary nanocomposites

**Figure 4:**
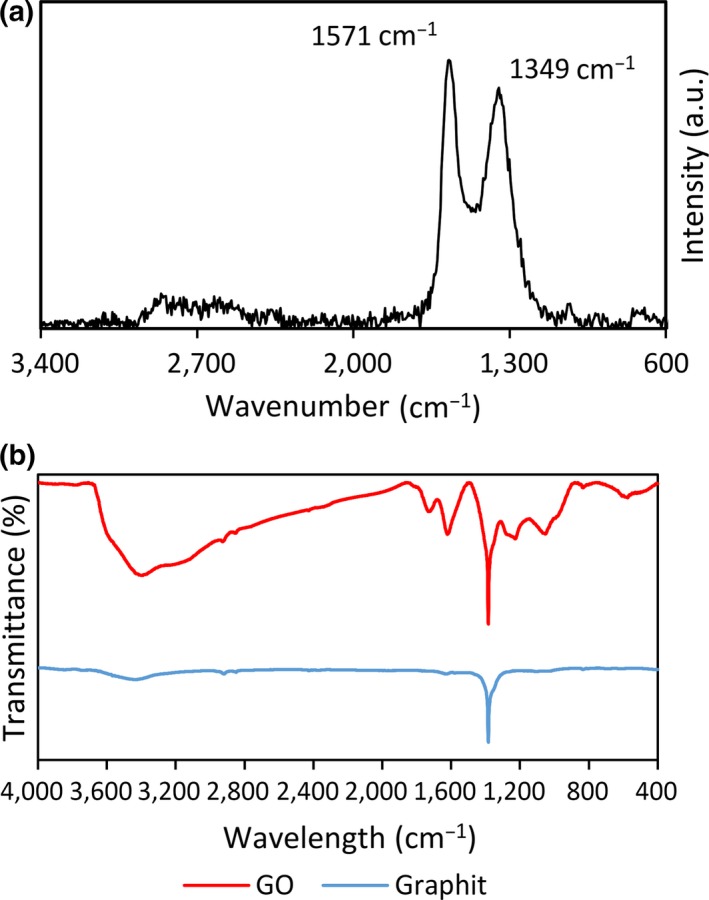
(a) Raman spectra of graphene oxide. Note the changes in intensity of D and G bands. (b) FTIR spectra of GO and graphite

As shown in Figure 4a, the Raman spectrum of GO displayed two prominent peaks at 1,349 and 1,571 cm^−1^, ascribing to the well‐documented D and G bands in which the former corresponds to the breathing mode of j‐point phonons of A1g symmetry and the latter demonstrates the first‐order scattering of the E2g phonons (Fan et al., 2016; Jabeen et al., 2013; Xing et al., 2016). Considering the Raman spectra, peak D demonstrates the sp^3^ defects, specifically the oxygenated groups. Besides, peak G depicts the C‐containing groups of the graphene sheets in which the disorder in sp^2^ carbon agents was detected by the Raman spectra. The ratio of D/G indicates the intensity of the two peaks. Accordingly, if the final value of D/G is higher than 1.5, then sheets are completely destructed showing several fractures, whereas the D/G value of less than 1.5 corresponds to the preservation of the main structure (Khenfouch et al., 2014). Based on the obtained D/G value of 0.85 which correlated to the size of the sp^2^ domains, our results revealed that the desired structure of the sheets was maintained and no damage was observed in the final architecture (Jabeen et al., 2013). As GO was reduced to graphene during borohydride reduction, defects induced by removable oxygenated groups were partly restored, and the value of D/G increased.

The FTIR spectra of GO and graphite are depicted in Figure 4b. As shown, the peak of 3,405 cm^−1^ presents the O‐H stretching which is considerably wide for GO; however, it is not present in the graphite structure. Next, the peaks of 1,727 and 1621 cm^−1^ which belong to the functional groups of C=O stretching vibration along with C=C aromatic (C‐C) stretching vibration (Yu et al., 2016). Another peak was found at 1,384 cm^−1^ for both structures, while in GO, the transmittance value was higher compared to graphite which corresponds to the presence of C‐O group (epoxy) and C‐O (alkoxy) (Wang et al., 2014). As previously mentioned, these bonds (C‐O group (epoxy) and C‐O (alkoxy)) are in contact with a rigid body inside the GO structure, so the stretching vibration value is intensified. Other major peaks detected in the FTIR spectra include 1,227 and 1,052 cm^−1^ corresponding to C‐O (epoxy) stretching that was absent in graphite and detected in GO structure (Xing & Wang, 2016).

### 3.4 Energy‐dispersive X‐ray (EDX) spectra and surface elemental mapping results

According to Figure 3, whenever the concentration of graphene oxide was higher than active carbon, higher level of oxygen was also expected (76.76%) and *vice versa* as we noticed the amount of oxygen was reduced to 49.47%. In sample C_400_‐G_400_, the concentration of graphene oxide was equal to active carbon; therefore, the level of oxygen was supposed to be between the samples C_300_‐G_600_ and C_600_‐G_300;_ however, the oxygen level was not higher than C_600_‐G_300_ and was totally different from our expectation which need to be explored.

As seen in the FE‐SEM images, the amount of zero‐valent iron should be reduced with respect to the reduction rate in graphene oxide. Also, in samples containing higher levels of active carbon in comparison to graphene oxide, it is expected that the iron level is between the two other samples, while the iron level was significantly increased. Furthermore, graphene oxide sheets and the rod shape of active carbon was visible in FE‐SEM image (Figure 3c, sample C_600_‐G_300_) Accordingly, active carbon plus iron nanoparticles were clearly observed as graphene oxide level was reduced. When GO concentration was higher than active carbon, nZVI was observed in the form of clusters and GO sheets. Also, active carbon was not observable as shown in Figure 3a (sample C_300_‐G_600_) which is in line with the relevant AFM image illustrating the ordered and intact nZVIs (Figure 2d). On the other hand, with respect to the equal concentration of these materials together with the EDS results, higher concentration of zero‐valent iron was observed.

It seems necessary to analyze the composite structure in order to optimize the availability level of the iron nanoparticles. As seen in Figure 1e, in the control sample when graphene oxide is not present, the level of zero‐valent iron is nearly 1.67% which is the minimum level compared to the time when graphene oxide is present in the composite, and therefore, the presence of graphene oxide is necessary for the adherence of iron in the composite structure.

**Figure 5:**
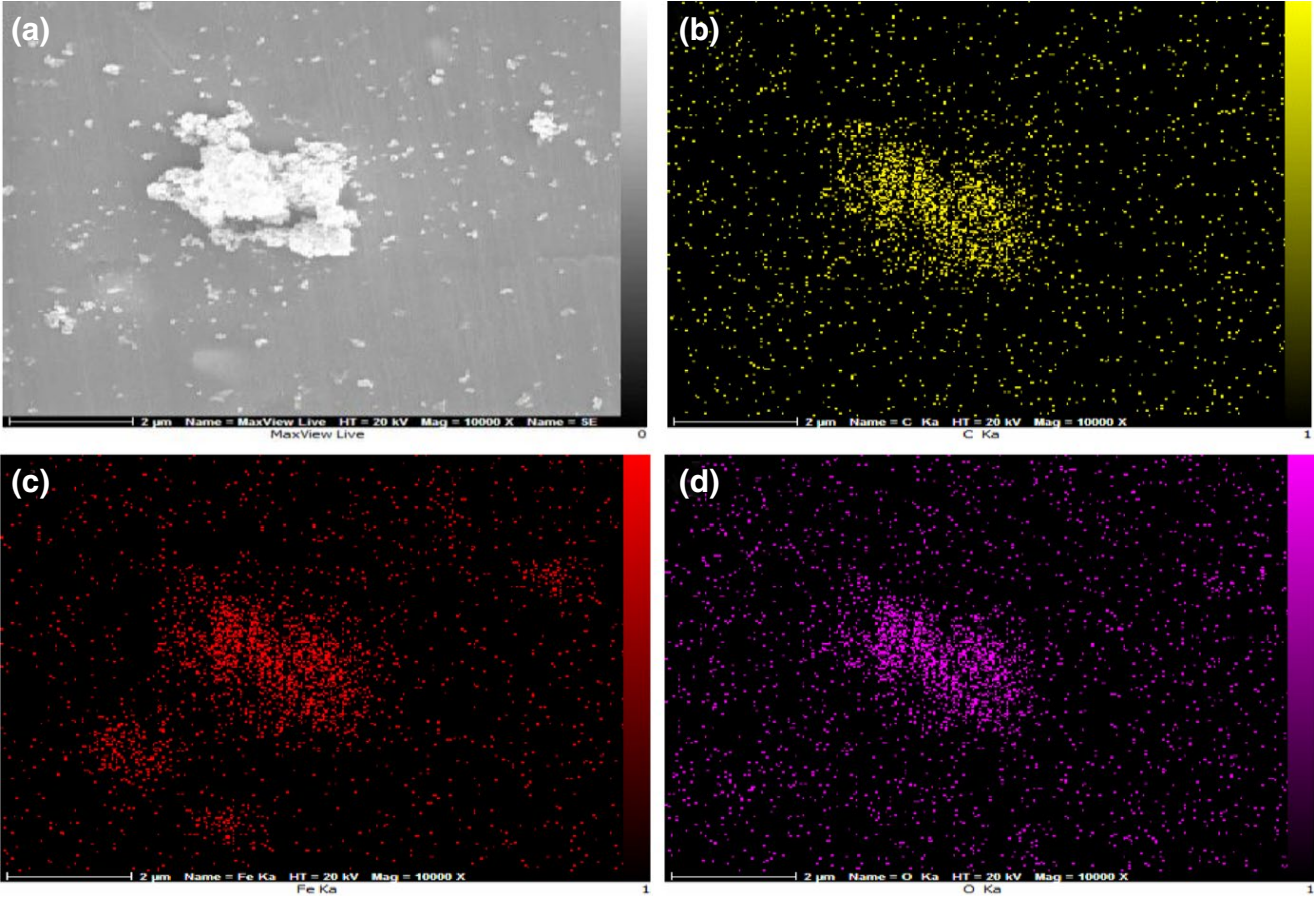
Surface mapping of sample C_600_‐G_300_: (a) FE‐SEM image, (b) carbon mapping, (c) iron mapping, and (d) oxygen mapping

Furthermore, the element mapping of C, Fe, and O is shown in Figure 5 analyzed by energy‐dispersive X‐ray spectroscopy in which all the elements are distributed in a homogenous pattern. In a similar work Sahu, Arora, Banik, Iyer, and Qureshi (2017) performed an elemental mapping test to detect the distribution pattern of Fe, O, C, and silicon in the GO composites.

## 4 CONCLUSION

nZVI has been employed to adsorb different types of compounds and pollutants. Active carbon and graphene oxide have also been used for adsorption and purification purposes. In this study, a combination of these materials was implemented to synthesize a nanocomposite/nanoadsorbent, and the structural along with the chemical characteristics of the fabricated nanocomposites was explored. Our results demonstrated that the preparation method leads to the formation of needle‐shaped active carbon bodies which enhances the available surface area for reactions. Moreover, with the help of ultrasonication, the space between graphene oxide sheets was enhanced which in turn accommodates more active carbon and nZVIs that can be used for adsorption processes. Also, the concentration of graphene oxide had an increasing effect on the deposition process and leads to the formation of homogeneous particle sizes. By investigating the structural properties via FTIR and Raman spectra, the formation of O‐H stretching groups plus the D and G bands showed that the structure of graphene oxide sheets maintained their stable structure. Overall, among the entire samples, sample C_600_‐G_300 _had the lowest GO content plus a higher nZVI content due to higher chance of the deposition of nZVI on the available surfaces. According to the studied structural and chemical attributes of the fabricated nanocomposite, it is suggested that this nanostructure can be employed as a robust compound for the nanoadsorption of different compounds, especially polyphenols found in the by‐products of food industry waste.

## CONFLICT OF INTEREST

All authors declare that there is no conflict of interest.

## ETHICAL STATEMENT

There was no human or animal testing in this study.
